# Advanced Prehospital Airway Management: Analyzing Success Rates and Predictors of King Laryngeal Tube Use

**DOI:** 10.3390/healthcare14131831

**Published:** 2026-06-24

**Authors:** Meshary S. Binhotan, Randa I. Almadhari, Ahmed M. Alotaibi, Abdulrhman S. Alghamdi, Meshal E. Alharbi, Abrar Almutairi, Abdullah N. Alshibani

**Affiliations:** 1 Emergency Medical Services Department, College of Applied Medical Sciences, King Saud Bin Abdulaziz University for Health Sciences, Riyadh 11481, Saudi Arabia; otaibia@ksau-hs.edu.sa (A.M.A.); ghamdia@ksau-hs.edu.sa (A.S.A.); harbim@ksau-hs.edu.sa (M.E.A.); alshibania@ksau-hs.edu.sa (A.N.A.); 2 King Abdullah International Medical Research Center, Riyadh 11481, Saudi Arabia; almutairiabr@ksau-hs.edu.sa; 3 Emergency Medical Services, Emergency Medicine Department, King Abdulaziz Medical City, Ministry of the National Guard-Health Affairs, Riyadh 11426, Saudi Arabia; 4 Research Department, Saudi Red Crescent Authority, Riyadh 11129, Saudi Arabia; r.i.almadhri@gmail.com; 5 Research Unit, College of Applied Medical Sciences, King Saud Bin Abdulaziz University for Health Sciences, Riyadh 11481, Saudi Arabia

**Keywords:** advanced airway management, king LT, king laryngeal tube, supraglottic airway, Saudi Arabia, prehospital care

## Abstract

**Background/Objectives**: Prehospital advanced airway management significantly affects patient outcomes. The King Laryngeal Tube (King LT) has been a standard method for managing compromised airways in various emergency medical services (EMSs). However, in-depth analyses of first-attempt success and influencing factors are limited. This study explores the use of the King LT in Saudi Arabia to assess the first-attempt success rate and predictors of successful management. **Methods**: This retrospective cross-sectional study was conducted to analyze cases requiring the King LT in the main EMS provider in Saudi Arabia between October 2021 and September 2022. A descriptive analysis was employed for categorical data, and Chi-square test, Fisher’s exact test, and a regression analysis were applied to assess the significance of the association. **Results**: Of the 239 analyzed cases, adults (58.6%) and males (70.7%) were predominant. The highest proportions of cases were medical cases (36.8%) and indoor incidents (69.9%), with a significant association of indoor incidents with female and elderly patients (*p* = 0.001). The first-attempt success rate reached 82.4%, with significant success likelihood in afternoon incidents (adjusted odds ratio [OR] = 2.92, 95% confidence interval [CI] [0.53–3.57]; *p* = 0.03). **Conclusions**: This first nationwide study of King LT outlines advanced airway management characteristics in Saudi Arabia. The high use rates in adults, males, medical cases, and indoor incidents could suggest tailored training strategies. Noted temporal variations may provide insights for policy improvements. While first-attempt success rates are high, reflecting literature findings; performance could improve with further training.

## 1. Introduction

Airway management is a crucial aspect of prehospital care for emergency medical service (EMS) providers [1,2]. Safe airway management is critical to maintaining oxygenation and ventilation, saving the lives of patients [1,2]. Critically ill and injured patients, including those with respiratory failure, compromised airways, and a low Glasgow Coma Scale (≤8), likely require advanced airway management prior to hospital arrival [3]. Moreover, EMS providers are trained to manage such cases within their scope of practice. Advanced airway management can include the use of endotracheal tubes (ETTs) and supraglottic airway (SGA) devices, such as a laryngeal mask airway, esophageal–tracheal Combitube, and the King laryngeal tube (King LT) [3].

The ETT has primarily been the method that EMS providers use in advanced airway management to secure compromised airways [1,4]. However, applying ETTs in a prehospital environment is challenging. The successful insertion of an ETT requires skills and regular training [1]. Further, EMS providers infrequently apply the ETT in clinical practice [5], resulting in a low and varied success rate [6,7]. Some studies have also revealed adverse events due to ETT insertion by paramedics [8,9,10,11]. The SGA, which is the traditional rescue method, provides an alternative option to manage the airway safely [12]. Over the last decade, the SGA has been used as a rescue method after unsuccessful ETT intubation and as a primary method of airway management in both hospital and prehospital settings [13]. Some evidence indicates similar benefits of using SGA devices compared with ETTs in prehospital settings, and could outweigh the benefits of the ETT due to the infrequent exposure of EMS providers to the ETT in clinical practice, increasing the risk of failed ETT intubations [14,15].

Among SGA devices, the King LT has been widely accepted and employed by many EMS institutions. The King LT is easy to use and requires less training and fewer skills than the ETT [16,17], making it a safe method to secure the airway in prehospital settings. The King LT also has shown a faster insertion time and higher success rate compared with other SGA devices [18,19] and ETT intubation [16,17]. However, in the literature, in-depth analyses of the successful first-attempt rate and factors influencing it for the King LT are limited.

Prehospital care in Saudi Arabia is primarily managed by the Saudi Red Crescent Authority (SRCA), which responds to all emergency calls, including those requiring advanced airway management, across Saudi Arabia. The SRCA and other Saudi hospital-based EMS providers are trained to deliver various types of airway management, including those requiring the King LT. However, studies exploring advanced airway management in prehospital settings are scarce. Only one study in Saudi Arabia has explored the successful insertion rate of the ETT and SGA, finding a 69% successful rate for ETT intubation and a 100% successful rate for the SGA [20]. However, this study had a small sample size of 16 patients for the ETT and eight for the SGA and was limited to only hospital-based EMS providers. Therefore, further studies with a larger sample size are recommended to explore advanced airway management.

The existing body of research on King LT regarding comprehensive analyses of first-attempt success rates and their influencing predictors is scarce, and no investigation in Saudi Arabia has yet addressed this topic. Therefore, this study aims to investigate the King LT success rate in Saudi Arabia and identify predictors of successful King LT insertion. The study’s findings may help understand advanced airway management performance and could provide insights to policymakers for potential improvement plans, such as targeted training programs.

## 2. Materials and Methods

### 2.1. Study Design and Setting

This cross-sectional retrospective study analyzes King LT management by SRCA providers from October 2021 to September 2022. The SRCA is a public governmental institution that provides prehospital care to citizens and residents throughout Saudi Arabia. This study assesses all cases where SRCA providers attempted to apply the King LT across Saudi Arabia. The SRCA completes an electronic patient care report for each patient, and the collected data were electronically extracted from the report using the SRCA system.

### 2.2. Data Source and Collection

This study analyzes 239 cases in which SRCA providers employed King LT interventions throughout the regions of Saudi Arabia. The data were electronically extracted using the SRCA system. This study includes:

- Patients from all age groups;
- Both genders;
- Patients who received King LT intervention in prehospital settings.

The exclusion criteria were:

- Patients who underwent King LT intervention in hospital settings;
- Duplicate patient records;
- Patients with incomplete documentations.

The extracted dataset underwent duplicate and completeness checks, ensuring only one record is available for each case, using a unique case identifier. Complete documentation of key variables (e.g., number of King LT attempts and patient’s age) in each case was assessed. Duplicates were removed from the final dataset, and no missing variables were identified.

### 2.3. Variables and Measurements

This study includes a comprehensive dataset to provide the King LT success rate and identify predictors of successful King LT management. Sociodemographic variables include age (pediatric: ≤17; adult: 18 to 64; and elderly: ≥65) and gender. Regional variables (city) and temporal variables, including day (weekdays: Sunday to Thursday, and weekends: Friday and Saturday), time (morning, afternoon, evening, and night), and month, were collected to detect regional and temporal patterns. The incident type (e.g., respiratory, cardiac, and trauma) and place (e.g., residential area, street, and workplace) were collected to provide contextual variables for the incidents. The use of rapid sequence intubation (RSI) medication was also documented, and the number of King LT attempts was recorded to determine the success rate.

### 2.4. Statistical Analysis

The statistical analysis was conducted using the Statistical Package for the Social Sciences (SPSS) for Windows (IBM Corp., Armonk, NY, USA) (v. 26.0). Descriptive statistics were employed to present sociodemographic data for the categorical variables, including the frequency and percentages. Chi-square test and Fisher’s exact test were applied to determine the association between gender and age with other characteristics, including the number of King LT attempts, incident type, patient location, time, and season. Effect size was measured using Phi coefficient for 2 × 2 tables and Cramer’s V for tables with three or more categories. Binary logistic regression analysis was employed to determine the association between the number of attempts and the variables adjusted for day, time, season, age, gender, incident type, and patient location. Model fit was assessed using the Hosmer–Lemeshow goodness-of-fit test. Statistical significance was set at *p* < 0.05.

### 2.5. Ethical Considerations

Ethical approval was received by the Institutional Review Board (IRB) at King Abdullah International Medical Research Center (NRC23R/702/11, 12 November 2023) and SRCA (22-26E, 22 December 2022), and the principles of the Declaration of Helsinki were adhered to. The IRB committees waived informed consent due to the retrospective nature of this study and the lack of direct human interaction. No personal or identifiable data were analyzed to require anonymity. All measures to ensure confidentiality and privacy were taken throughout this study, and all data were stored on a password-protected computer with access limited to only research team members.

## 3. Results

**Figure 1:**
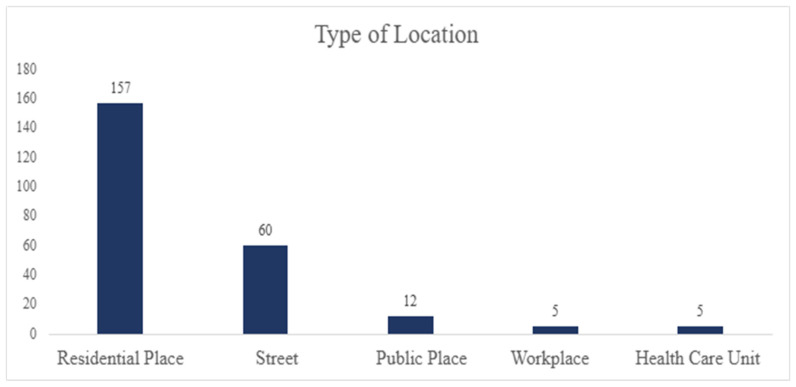
Detailed analysis of the incident location requiring King laryngeal tube airway management.

This study analyzes 239 patient cases, with the adult age group (58.6%) being predominant (Table 1). The male group accounted for most of the sample, at 70.7%. Most patients were from Riyadh (29.3%), followed by Jeddah (24.6%). The most common incident type was medical (36.8%), followed by arrest (29.7%), whereas drowning cases were the least common (2.5%). Indoor incidents (69.9%) occurred more often than outdoor incidents (30.1%). Residential areas ranked the highest for incidents (65.7%), followed by streets (25.1%), whereas public places and healthcare units ranked lowest (5.0%; Figure 1). King LT insertion was successful on the first attempt in most cases (82.4%), with the rare use of RSI medications (2.1%).

Table 2 depicts the seasonal and temporal patterns of the incidents, highlighting that most incidents occur at night (34.7%) and during weekdays (66.9%). Incidents peaked on Friday (17.6%) and dropped on Monday (7.5%). The seasonal analysis revealed variations, with the highest proportion of cases occurring in summer (33.5%) and the least in autumn (13.8%).

Table 3 presents the association between gender and incident-related variables, revealing significant gender differences. Successful first-attempt King LT insertion was higher for females (87.1%) than for males (81.1%). Medical cases dominated the incident types among both males (36.1%) and females (38.6%), followed by arrest (males: 27.2%; females: 35.7%). However, trauma cases were double for males (20.7%) compared with females (10.0%). Indoor incidents were significantly higher for females (88.6%) than for males (62.1%), with outdoor incidents among males (37.9%) reported to be more than three times higher than those for females (11.4%; *p* = 0.001). Most incidents for the male group occurred at night (36.7%), whereas incidents for the female group were mostly reported in the morning (31.4%). The cases peaked during summer for both males (32.0%) and females (37.1%) and were lowest during autumn (male: 16.6%; female: 7.1%).

Table 4 indicates the association between age and other incident-related factors. The elderly group had the most successful first-attempt King LT insertion (88.1%), and the pediatric and adult groups had similar first-attempt success (80.0%). Medical incidents had the highest rate of cases among the elderly group (40.5%), whereas drowning and trauma were highest among the pediatric group (26.7%). Indoor incidents were high among the elderly group (94.0%), and outdoor cases were higher among the pediatrics (46.7%; *p* = 0.001). Night cases were higher in the adult group (41.4%), whereas afternoon cases peaked among the elderly group (33.3%), and morning cases were predominant in the pediatric group (46.7%; *p* = 0.012). The seasonal analysis detected higher cases during summer for the adult group (38.6%), during winter for the elderly group (34.5%), and during winter and spring for the pediatric group (33.3%).

Table 5 presents the regression analysis of successful first-attempt King LT insertions by age, gender, incident type, time of the day, season, type of the day, and patient location. Afternoon incidents were 2.92 times more likely to have a successful first attempt compared to night incidents (95% confidence interval [CI] [0.53–3.57]; *p* = 0.03). The other variables showed no significant associations. Hosmer–Lemeshow goodness-of-fit test indicated adequate model fit (χ^2^ = 5.217, df = 8, *p* = 0.734).

## 4. Discussion

This study assessed the use of the King LT to identify the first-attempt success rate and the predictors of successful insertion, providing the first assessment of advanced airway management using the King LT nationwide in Saudi Arabia. Most patients were adult and male, highlighting the high risk of these groups. Indoor incidents and medical causes were the predominant reasons for using the King LT. The successful first-attempt rate reached 82.4%, reflecting similar findings in the literature and supporting continuous training programs. Afternoon incidents displayed a significant association with the successful first attempt. The findings of this investigation might offer insights that could guide future targeted education programs for paramedics and potentially provide guidance for improving prehospital advanced airway management protocols.

The findings revealed a high first-attempt success rate (82.4%), reflecting similar findings in the broader literature. A study in the United States that reviewed all King LT interventions in 35 ground EMS agencies and air medical critical care services revealed that paramedics and prehospital nurses performed first attempts successfully at 84.9% [13]. Other studies noted similar findings, with success rates ranging between 81% and 88% [12,17,19]. The slight differences in the success rate could be due to the differences in the underlying etiologies and demographics of the included patients, such as the differences in age groups [7]. Despite these rates being considered high, especially compared to the use of the ETT [21], training and real-case exposure could enhance this rate. This skill is significant as successful airway placement is a quality measure for advanced airway management performance [7,22], and a first-attempt success yields better patient outcomes [23].

The male (70.7%) and adult (58.6%) groups were the predominant recipients of the King LT, highlighting their high risk of being involved in emergency cases. This finding aligns with those of international studies examining advanced airway management in prehospital services [7,13,24]. A large-scale study of 32,592 patients across 16 states in the US who received various types of advanced airway intervention revealed a predominance of male adults as the recipients [24]. This high risk could be attributed to the epidemiological profile of this group and their greater engagement in high-risk activities and occupational exposure, requiring prehospital care and intervention [25].

The analysis identified indoor medical cases as the predominant reason for advanced airway intervention, mirroring the typical characteristics of advanced airway interventions in the wider literature. These characteristics were also noted in the national Out-of-Hospital Cardiac Arrest (OHCA) Registry data in Saudi Arabia [26]. Several studies have also highlighted the highest application of advanced airway management, including the King LT, among patients experiencing cardiac arrest and medical emergencies, with indoor settings being a common location for these cases [27,28,29,30]. This finding could support customized training to simulate indoor settings as these settings might make airway procedures more challenging due to the potentially limited access to the patient, space, and lighting [31]. Among both genders, female patients were more likely than male patients to experience an indoor incident (88.6% vs. 62.1%, *p* = 0.001), reflecting global findings in which the female group was involved in a higher proportion of indoor critical cases requiring advanced airway management compared with the male group [32]. Despite indoor incidents dominating across all age groups, almost all elderly group cases (94.0%) occurred indoors, likely due to comorbidities and reduced mobility, limiting outdoor activities [33]. These findings present a demographic profile of King LT interventions, potentially providing insights for tailored training programs for paramedics.

The temporal analysis revealed meaningful associations across age groups (*p* = 0.012) and factors influencing the successful first attempt of King LT use. Night incidents were more frequent for the adult group (41.4%), while afternoon incidents were highest among the elderly group (33.3%), and morning incidents were most common for the pediatric group (46.7%). These temporal variations across age groups were also noted in another study investigating OHCA incidents [34]. Afternoon incidents were more likely to result in first-attempt success than night incidents (adjusted OR = 2.92, 95% CI [0.53–3.57]; *p* = 0.03), mirroring similar findings in which previous OHCA registry studies have reported poorer outcomes at night [35,36]. A Polish cardiac arrest registry analysis also found lower return of spontaneous circulation at night compared with daytime cases [37]. Although this study did not assess survival outcomes, the findings support the view that nighttime events may occur under conditions challenging the provision of prehospital care, including resuscitation and airway management. Further evidence showed decreased alertness, slower reaction times, and worse decision-making during night shifts, particularly with cumulative sleep restriction and circadian disturbances, providing possible justification for the higher success rate in the afternoon compared to at night [38]. The lower first-attempt success rate at night may also indicate staffing patterns and fatigue, which might suggest the need for targeted training and operational support for overnight crews.

### 4.1. Study Limitations

This study has some limitations that need to be acknowledged. The retrospective design limits the ability to follow up with these patients to assess the influence of the King LT intervention on patient outcomes. Additionally, the nature of the observational cross-sectional design prevents conclusions about causal relationships. Furthermore, the relatively low number of failed events (*n* = 41) compared to successful first-attempt cases may affect the accuracy of the significance of the findings. However, the findings reported are based on data collected from real cases, demonstrating that most cases were successful on the first attempt. This may also limit the precision of odds ratio estimates, as reflected in the wide confidence intervals observed for some variables, particularly for season and age categories. Finally, the lack of qualitative findings hinders the discovery of the actual reasons for failed King LT attempts.

### 4.2. Recommendations for Future Research

The findings of this study could suggest directions for future research. Interviewing EMS providers to explore their views and opinions about challenges and facilitators in performing a King LT intervention and collecting detailed contextual data could inform further improvement strategies, enhancing the success rate of using the King LT. Additionally, conducting a study incorporating patient outcomes and follow-up metrics is recommended to assess the influence of successful and failed King LT interventions. Lastly, undertaking research with a larger sample size is recommended to provide more precise estimates of the findings and confirm this study’s results.

## 5. Conclusions

This study presents an analysis of the King LT performance nationwide in Saudi Arabia, providing a characteristic profile of advanced airway management. The first-attempt success rate reached 82.4%, mirroring similar findings in the literature; therefore, further training programs are recommended to improve this rate. The male and adult groups were at high risk, and indoor medical incidents dominated the cases. The first-attempt success rate was significantly associated with afternoon incidents compared to night incidents. These findings may offer insights to policymakers about the potential need for tailored training programs and considerations for operational strategy adjustments to adapt to these variations. However, further qualitative analyses to capture the opinions of EMS providers and assess contextual factors could improve our understanding of the facilitators and challenges of the King LT.

## Figures and Tables

**Table 1:** Patient Demographics and Case Characteristics for King Laryngeal Tube Airway Management.

Variable	*N* (%)
**Age**	
Pediatric (≤17)	15 (6.3)
Adult (18–64)	140 (58.6)
Elderly (≥65)	84 (35.1)
**Gender**	
Male	169 (70.7)
Female	70 (29.3)
**City**	
Arar	9 (3.8)
Asir	6 (2.5)
Dammam	6 (2.5)
Hail	2 (0.8)
Jizan	5 (2.1)
Al-Jouf	5 (2.1)
Madinah	36 (15.1)
Makkah	63 (24.6)
Najran	23 (9.6)
Al-Qassim	3 (1.9)
Riyadh	70 (29.3)
Tabuk	11 (4.6)
**Incident type**	
Respiratory	22 (9.2)
Arrest	71 (29.7)
Cardiac	7 (2.9)
Medical	88 (36.8)
Trauma	42 (17.6)
Drowning	6 (2.5)
Other	3 (1.3)
**Patient location**	
Indoor	167 (69.9)
Outdoor	72 (30.1)
**Number of attempts**	
First attempt	198 (82.4)
Second attempt	41 (17.6)
**RSI medications ^1^**	
Yes	5 (2.1)
No	234 (97.9)

^1^ Rapid Sequence Intubation (RSI).

**Table 2:** Temporal and Seasonal Analysis of Cases of King Laryngeal Tube Airway Management.

Variable	*N* (%)
**Day**	
Saturday	37 (15.5)
Sunday	31 (13.0)
Monday	18 (7.5)
Tuesday	39 (16.3)
Wednesday	34 (14.2)
Thursday	38 (15.9)
Friday	42 (17.6)
**Type of day**	
Weekday	160 (66.9)
Weekend	79 (33.1)
**Time of the day**	
Morning	67 (28.0)
Afternoon	53 (22.2)
Evening	36 (15.1)
Night	83 (34.7)
**Season**	
Spring	53 (22.2)
Summer	80 (33.5)
Autumn	33 (13.8)
Winter	73 (30.5)

**Table 3:** Association Between Gender and Incident Characteristics.

Variables	Gender		X2	df	*p*-Value	Effect Size
	Male *N* (%)	Female *N* (%)				
**Number of attempts**						
First attempt	137 (81.1)	61 (87.1)	1.28	1	0.34	Phi = 0.073
Second attempt	32 (18.9)	9 (12.9)				
**Incident type**						
Respiratory	15 (8.9)	7 (10.0)	9.09	6	0.14	Gramer’s V = 0.19
Arrest	46 (27.2)	25 (35.7)				
Cardiac	5 (3.0)	2 (2.9)				
Medical	61 (36.1)	27 (38.6)				
Trauma	35 (20.7)	7 (10.0)				
Drowning	6 (3.6)	0 (0.0)				
Other	1 (0.6)	2 (2.9)				
**Patient location**						
Indoor	105 (62.1)	62 (88.6)	16.4	1	0.001	Phi = 0.262
Outdoor	64 (37.9)	8 (11.4)				
**Type of day**						
Weekday	111 (65.7)	49 (70.0)	0.41	1	0.31	Phi = 0.04
Weekend	58 (34.3)	21 (30.0)				
**Time of the day**						
Morning	45 (26.6)	22 (31.4)	1.92	3	0.58	Gramer’s V = 0.09
Afternoon	35 (20.7)	18 (25.7)				
Evening	27 (16.0)	9 (12.9)				
Night	62 (36.7)	21 (30.0)				
**Season**						
Spring	36 (21.3)	17 (24.3)	3.80	3	0.28	Gramer’s V = 0.12
Summer	54 (32.0)	26 (37.1)				
Autumn	28 (16.6)	5 (7.1)				
Winter	51 (30.2)	22 (31.4)				

Chi-square test was used for all categorical comparisons. Phi coefficient reported for 2 × 2 tables; Cramer’s V reported for tables with 3 or more categories. Significance level set at *p* < 0.05.

**Table 4:** Association Between Age and Incident Characteristics.

Variables	Age			X2	df	*p*-Value	Effect Size
	Pediatric *N* (%)	Adult *N* (%)	Elderly *N* (%)				
**Number of attempts**							
First attempt	12 (80.0)	112 (80.0)	74 (88.1)	2.51	2	0.27	Gramer’s V = 0.10
Second attempt	3 (20.0)	28 (20.0)	10 (11.9)				
**Incident type name**							
Respiratory	2 (13.3)	10 (7.1)	10 (11.9)	62.5	12	0.12	Gramer’s V = 0.36
Arrest	4 (26.7)	34 (24.3)	33 (39.3)				
Cardiac	0 (0.0)	4 (2.9)	3 (3.6)				
Medical	1 (6.7)	53 (37.9)	34 (40.5)				
Trauma	4 (26.7)	35 (25.0)	3 (3.6)				
Drowning	4 (26.7)	2 (1.4)	0 (0.0)				
Other	0 (0.0)	2 (1.4)	1 (1.2)				
**Patient location**							
Indoor	8 (53.3)	80 (57.1)	79 (94.0)	36.0	2	0.001	Gramer’s V = 0.38
Outdoor	7 (46.7)	60 (2.7)	5 (6.0)				
**Type of day**							
Weekday	10 (66.7)	89 (63.6)	61 (72.6)	1.94	2	0.37	Gramer’s V = 0.09
Weekend	5 (33.3)	51 (36.4)	23 (27.4)				
**Time of the day**							
Morning	7 (46.7)	34 (24.3)	26 (31.0)	16.3	6	0.012	Gramer’s V = 0.18
Afternoon	2 (13.3)	23 (16.4)	28 (33.3)				
Evening	2 (13.3)	25 (17.9)	9 (10.7)				
Night	4 (26.7)	58 (41.4)	21 (25.0)				
**Season**							
Spring	5 (33.3)	23 (16.4)	25 (29.8)	11.3	6	0.07	Gramer’s V = 0.15
Summer	4 (26.7)	54 (38.6)	22 (26.2)				
Autumn	1 (6.7)	24 (17.1)	8 (9.5)				
Winter	5 (33.3)	39 (27.9)	29 (34.5)				

Chi-square test was used for all categorical comparisons. Cramer’s V was reported as effect size measure. Significance level set at *p* < 0.05.

**Table 5:** Regression Analysis of Successful First-Attempt King Laryngeal Tube Insertion Factors.

Variables	Crude OR (95% CI)	*p*-Value	Adjusted OR (95% CI)	*p*-Value
**Age**				
Adult	Ref	Ref	Ref	Ref
Pediatric	1.0 (0.26–3.78)	1.0	0.67 (0.15–2.85)	0.59
Geriatric	0.54 (0.24–1.17)	0.12	0.42 (0.08–2.00)	0.27
**Gender**				
Male	Ref	Ref	Ref	Ref
Female	0.63 (0.28–1.40)	0.26	0.70 (0.28–1.74)	0.44
**Incident type**				
Non-arrest	Ref	Ref	Ref	Ref
Arrest	1.12 (0.54–2.31)	0.75	1.28 (0.5–2.86)	0.54
**Time of the day**				
Morning	1.25 (0.50–3.10)	0.62	1.5 (0.59–4.23)	0.35
Afternoon	2.36 (0.94–5.96)	0.06	2.92 (0.53–3.57)	0.03
Evening	0.62 (0.23–1.67)	0.35	0.72 (0.26–2.02)	0.54
Night	Ref	Ref	Ref	Ref
**Season**				
Spring	1.51 (0.60–3.82)	0.37	1.48 (0.55–4.01)	0.43
Summer	2.44 (0.85–7.03)	0.09	2.40 (0.78–7.34)	0.12
Autumn	0.50 (0.16–1.55)	0.23	0.47 (0.14–1.58)	0.22
Winter	Ref	Ref	Ref	Ref
**Type of day**				
Weekday	0.60 (0.27–1.30)	0.19	0.64 (0.28–1.47)	0.30
Weekend	Ref	Ref	Ref	Ref
**Patient location**				
Indoor	Ref	Ref	Ref	Ref
Outdoor	1.62 (0.80–3.26)	0.17	1.04 (0.44–2.41)	0.92

Binary logistic regression analysis. OR = Odds Ratio; CI = Confidence Interval. Significance level set at *p* < 0.05.

## Data Availability

The data presented in this study are available upon reasonable request from the corresponding author, with permission from the Saudi Red Crescent Authority. These data belong to the Saudi Red Crescent Authority and were collected for the purpose of this study; therefore, prior permission is required.

## Abbreviations

The following abbreviations are used in this manuscript:

EMS	Emergency medical service
ETT	Endotracheal tube
SGA	Supraglottic airway
King LT	King laryngeal tube
SRCA	Saudi Red Crescent Authority
RSI	Rapid sequence intubation
SPSS	Statistical Package for the Social Sciences
IRB	Institutional Review Board
OHCA	Out-of-Hospital Cardiac Arrest
